# Variation in Stability of Endogenous Reference Genes in Fallopian Tubes and Endometrium from Healthy and Ectopic Pregnant Women

**DOI:** 10.3390/ijms13032810

**Published:** 2012-03-02

**Authors:** Alpha K. Gebeh, Emma L. Marczylo, Akwasi A. Amoako, Jonathon M. Willets, Justin C. Konje

**Affiliations:** 1 Endocannabinoid Research Group, Reproductive Science Section, Department of Cancer Studies and Molecular Medicine, University of Leicester, Leicester, LE2 7LX, UK; E-Mails: ag271@le.ac.uk (A.K.G.); aaa57@le.ac.uk (A.A.A.); jmw23@le.ac.uk (J.M.W.); 2 Systems Toxicology, MRC Toxicology Unit, Hodgkin Building, University of Leicester, Lancaster Road, Leicester, LE1 9HN, UK; E-Mail: elt14@le.ac.uk

**Keywords:** ectopic pregnancy, fallopian tubes, endometrium, reference genes, geNorm, NormFinder, quantitative real time PCR, NAPE-PLD

## Abstract

RT-qPCR is commonly employed in gene expression studies in ectopic pregnancy. Most use RN18S1, β-actin or GAPDH as internal controls without validation of their suitability as reference genes. A systematic study of the suitability of endogenous reference genes for gene expression studies in ectopic pregnancy is lacking. The aims of this study were therefore to evaluate the stability of 12 reference genes and suggest those that are stable for use as internal control genes in fallopian tubes and endometrium from ectopic pregnancy and healthy non-pregnant controls. Analysis of the results showed that the genes consistently ranked in the top six by geNorm and NormFinder algorithms, were UBC, GAPDH, CYC1 and EIF4A2 (fallopian tubes) and UBC and ATP5B (endometrium). mRNA expression of NAPE-PLD as a test gene of interest varied between the groups depending on which of the 12 reference genes was used as internal controls. This study demonstrates that arbitrary selection of reference genes for normalisation in RT-qPCR studies in ectopic pregnancy without validation, risk producing inaccurate data and should therefore be discouraged.

## 1. Introduction

Ectopic pregnancy is a common early pregnancy complication in which the embryo implants outside the uterus. It is estimated to complicate 2% of pregnancies and, more importantly, is still associated with significant morbidity and mortality in many parts of the world [1,2]. The most common site is the fallopian tube (98.3%) with sites such as the ovary, cervix and abdomen only being rarely affected [3]. Sadly, the mechanisms that underlie tubal implantation are still not fully understood despite the expansion in ectopic pregnancy related research over the last decade [4]. Over the years, various tools have been employed to elucidate these mechanisms with one such tool being quantitative real time PCR (RT-qPCR).

RT-qPCR is an important and common technique used to examine changes in gene expression levels in various pathological states. It offers an advantage over other methods such as northern blotting because of its high sensitivity, specificity, ease of use and broad dynamic range [5,6]. This feature albeit advantageous comes with a price *i.e.*, that it is susceptible to variation in extraction and experimental processes including differences in RNA extraction, presence of contaminating DNA, efficiency of cDNA synthesis *etc.* [7–9]. These inherent vulnerabilities make normalisation of RT-qPCR data an absolute requirement in the interpretation of experimental data. One of the most common methods of normalisation is the use of a reference gene whose expression should remain constant under various experimental and disease conditions [10]. This compensates for variation in the experimental conditions since both the gene of interest and the reference gene are exposed to the same experimental conditions. However, in practice, it is unlikely that a perfect reference gene exists since several studies have demonstrated that reference genes themselves can be influenced by drugs, chemicals and disease states [9]. Therefore, the validity of gene expression results generated by this method of normalisation is highly dependent on the optimal selection of endogenous reference genes to which the genes of interest are to be normalised.

In gene expression studies on ectopic pregnancy, the most commonly used reference genes are β-actin (ACTB), glyceraldehyde-3-phosphate dehydrogenase (GAPDH) and RNA 18S ribosomal 1 (RN18S1). These genes are often selected arbitrarily without assessing their stability and suitability for use as reference genes despite the recommendations from the MIQE (Minimum Information for publication of Quantitative real time PCR Experiments) guidelines that justification of the choice and number of reference genes should be an essential part of RT-qPCR experiments [11]. This widespread practice risks producing inaccurate experimental data and should be discouraged. A study of the suitability of endogenous reference genes for relative gene expression studies in fallopian tubes and endometrium from ectopic pregnancy is lacking. The aims of this study were therefore to evaluate the suitability of 12 commonly used reference genes and to determine which of them are suitable genes for normalization of quantification of mRNA expression in fallopian tubes and endometrium from women with ectopic pregnancy as well as in non-pregnant women. The candidate genes evaluated were those from the human geNorm™ reference gene kit (PrimerDesign, Southampton, UK) *i.e.*, ACTB, ATP synthase, H^+^ transporting, mitochondrial F1 complex, beta polypeptide (ATP5B), Beta-2-microglobulin (B2M), Cytochrome c-1 (CYC1), Eukaryotic translation initiation factor 4A isoform 2 (EIF4A2), GAPDH, Tyrosine 3-monooxygenase/tryptophan 5-monooxygenase activation protein, zeta polypeptide (YWHAZ), Ubiquitin C (UBC), Ribosomal protein L13a (RPLA13), Succinate dehydrogenase complex subunit A flavoprotein (SDHA), Topoisomerase (DNA) 1 (TOP1), and RN18S1. We also evaluated the effects of using each of these genes on the expression of a test gene of interest in order to demonstrate the effects (if any) that arbitrary selection of reference genes may have on data interpretation. For this aspect of the study, the gene expression of *N*-acylphosphatidyl-ethanolamide phospholipase D (NAPE-PLD) in human endometrium was used. The choice of NAPE-PLD is partly due to our group’s interest in evaluating the role of the endocannabinoid system in early mammalian reproduction including tubal transport of embryos. For further details on NAPE-PLD and the endocannabinoid system and their role in tubal transport and early pregnancy, see reviews by Taylor *et al.* [12], Bambang *et al.* [13] and Gebeh *et al.* [4].

## 2. Results and Discussion

### 2.1. Patient Characteristics

There was no significant difference in age (yrs) in both the follicular (*n* = 4) and luteal (*n* = 4) phase control groups. However, as expected, the age of the non-pregnant group was significantly higher than that of the ectopic group (*n* = 4). The mean ± SD was 43.00 ± 6.27, (follicular), 41.25 ± 4.43 (luteal) and 28.75 ± 2.87 (ectopic pregnancy), *p* = 0.004 (one way ANOVA). There was no significant difference in BMI (kg/m^2^) between the three groups studied. The mean ± SD were as follows: 27.00 ± 4.97 (follicular), 29.38 ± 9.86 (luteal) and 27.50 ± 4.40 (ectopic pregnancy), *p* = 0.877 (one way ANOVA).

### 2.2. General Expression Levels of Endogenous Reference Genes

**Figure 1:**
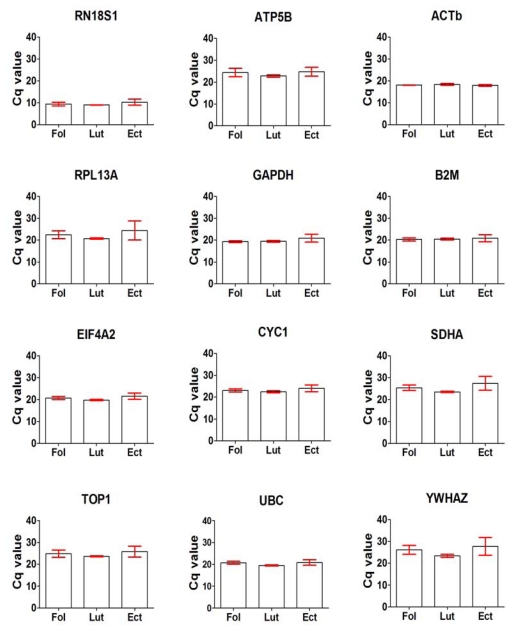
Expression levels of the 12 reference genes evaluated as shown by their Cq values in 12 fallopian tube samples. Samples were from the follicular (Fol, *n* = 4), luteal (Lut, *n* = 4) phase and ectopic pregnancy (Ect, *n* = 4). Cq values were obtained from the data output file of ABI PRISM 7000 and are inversely proportional to amount of mRNA template material present.

**Figure 2:**
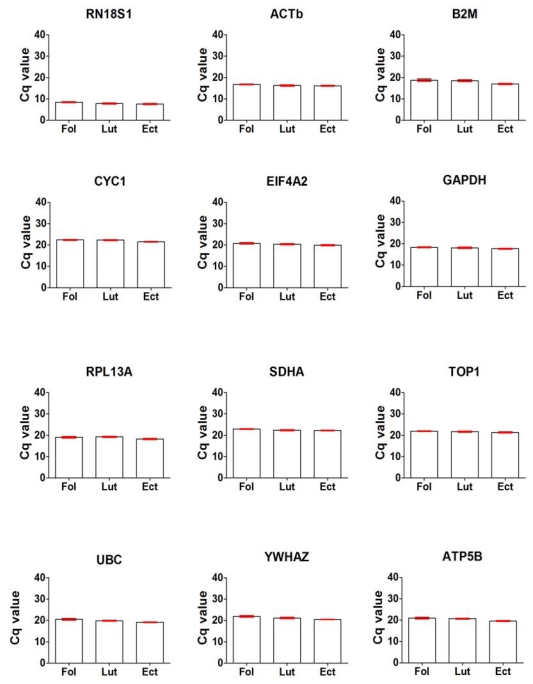
Expression levels of the 12 reference genes evaluated as shown by their Cq values in 12 endometrial samples. Samples were from the follicular (Fol, *n* = 4), luteal (Lut, *n* = 4) phase and ectopic pregnancy (Ect, *n* = 4). Cq values were obtained from the data output file of ABI PRISM 7000 and are inversely proportional to amount of mRNA template material present.

Overall, comparison of the Cq values demonstrated a wide variation within and between the groups especially the fallopian tube samples as shown in Figure 1. For fallopian tube samples, variation within the group was wider in ectopic pregnancy compared to those in the follicular and luteal phases, while for endometrial samples (Figure 2) within group variation was comparable in all the three study groups. There was a wide range in Cq values from an overall mean ± SEM (*n* = 12) of 9.61 ± 0.51 (RN18S1) to 25.6 ± 1.49 (YWHAZ) in fallopian tubes. This was also similar in the endometrial samples with an overall mean ± SEM (*n* = 12) of 7.94 ± 0.16 (RN18S1) to 22.55 ± 0.11 (SDHA).

### 2.3. Analysis of Reference Gene Stability in Fallopian Tubes

#### 2.3.1. geNorm Algorithm

In order to select the most suitable gene for normalisation, geNorm ranks the reference genes based on their expression stability value (*M*) with the highest *M* value equating to the least stable and *vice versa*. The cut-off for an acceptable reference gene is one with an *M* value of less than 1.5. The ranking of the reference genes evaluated starting from the most stable (lowest *M* value) to the least stable (highest *M* value) for these experiments were as follows: EIF4A2/CYC1 > UBC > RN18S1 > B2M > GAPDH > TOP1 > ATP5B > ACTB > SDHA > YWHAZ > RPL13A (Table 1). The *M* value for YWHAZ and RPL13A were greater than 1.5 indicating that they are unsuitable for use as internal control genes in fallopian tubes.

**Figure 3:**
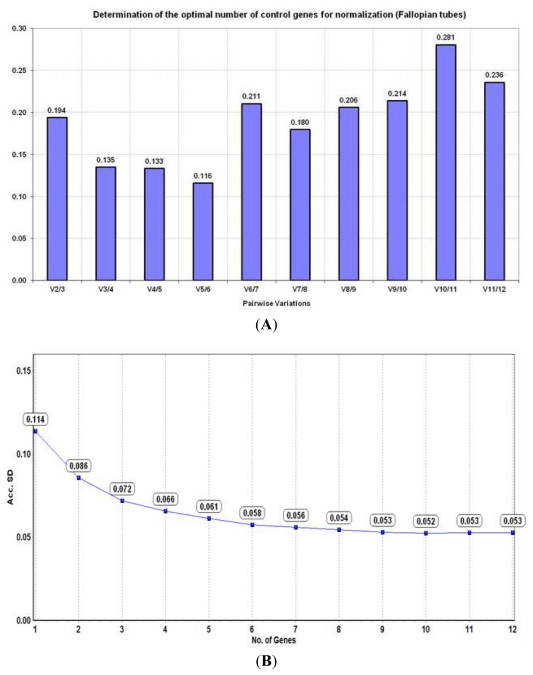
Determination of the optimal number of reference genes for normalization in fallopian tubes (*n* = 12) based on their (**A**) pairwise variation (V) analysis using the geNorm algorithm and (**B**) accumulated standard deviation (Acc. SD) using the NormFinder algorithm.

geNorm also calculates the pairwise variation between two normalisation factors (NF_n_ and NF_n+1_ genes) which gives an indication of the optimal number of reference genes to be used for appropriate normalisation of RT-qPCR data. A pairwise variation value of ≤0.15 is the acceptable cut-off below which the addition of an extra reference gene does not confer any advantage over the existing genes. Although EIF4A2 and CYC1 were the two best genes, their pairwise variation value was greater than 0.15 (V_2/3_ = 0.194) indicating that additional genes are required for adequate normalisation. Based on analysis by geNorm, EIF4A2, CYC1 and UBC (V_3/4_ = 0.135) was considered the minimum combination suitable for normalisation though theoretically, the best results would be obtained using a combination of 6 genes (V_5/6_ = 0.116) (Figure 3).

#### 2.3.2. NormFinder Algorithm

NormFinder uses a different mathematical model to geNorm and takes into account the intragroup and intergroup variation. In addition, NormFinder calculates a stability value for each reference gene. The most stable reference genes are those with the least inter- and intragroup variation and have the lowest stability value. The samples were divided into follicular (*n* = 4) and luteal phase (*n* = 4) and ectopic pregnancy (*n* = 4) for appropriate analysis by NormFinder. The ranking of the reference genes were as follows (starting from the most stable to the least stable): UBC > TOP1 > RPL13A > CYC1 > GAPDH > EIF4A2 > ATP5B > RN18S1 > B2M > YWHAZ > SDHA > ACTB (Table 1). The most suitable combination of genes was UBC and TOP1 (combined stability value = 0.064) which was different to that obtained using geNorm. Manual inspection of the data suggests that SDHA and YWHAZ had a wide intragroup variation while SDHA, YWHAZ and RPL13A had wide intergroup variation making them less suitable as reference genes.

The accumulated standard deviation (Acc. SD) was calculated using NormFinder and is an indicator of the optimal number of reference genes to be used for data normalisation. The lowest Acc. SD (theoretically indicating the optimal number of reference genes) was obtained when 10 genes were used (Acc. SD = 0.052) (Figure 3). However, TOP1 and UBC were selected by NormFinder as the best pair of genes for normalisation in fallopian tubes (Table 1).

### 2.4. Analysis of Reference Gene Stability in Endometrium

#### 2.4.1. geNorm Analysis

The ranking of the reference genes in the endometrium was different to that of the fallopian tubes and were as follows (starting from the most stable to the least stable): YWHAZ/UBC > ATP5B > SDHA > RN18S1 > EIF4A2 > TOP1 > CYC1 > ACTB > GAPDH > RPL13A > B2M (Table 2). Unlike the fallopian tubes, the *M* value for each of the 12 genes was less than 1.5 indicating that they were all reasonably stable enough to be used as reference genes in the endometrium.

**Figure 4:**
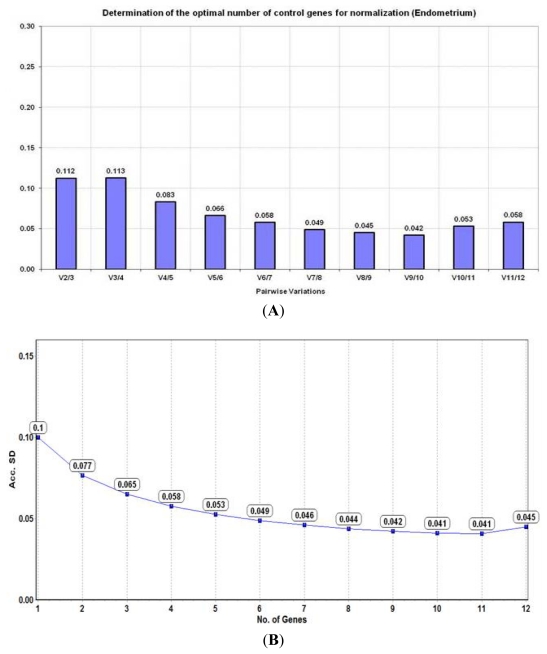
Determination of the optimal number of reference genes for normalisation in the endometrium (*n* = 12) based on their (**A**) pairwise variation (V) analysis using the geNorm algorithm and (**B**) accumulated standard deviation (Acc. SD) using the NormFinder algorithm.

Pairwise variation calculated by geNorm indicated that the combination of the top two reference genes in the endometrium *i.e.*, UBC and YWHAZ were stable enough to be used for normalisation without the addition of a 3rd gene (V_2/3_ = 0.112) (Figure 4). Although this was acceptable (V ≤ 0.15), the lowest pairwise variation score was obtained with a combination of 10 candidate genes (V_9/10_ = 0.042).

#### 2.4.2. NormFinder Algorithm

The ranking of the reference genes based on their stability values were as follows (starting from the most stable to the least stable): CYC1 > ATP5B > UBC> B2M > TOP1 > GAPDH > SDHA > YWHAZ > ACTB > RN18S1 > EIF4A2 > RPL13A (Table 2).

The lowest Acc. SD (0.041) was obtained using a combination of 10 reference candidates for normalisation (Figure 4). However, the most suitable combination of genes recommended by NormFinder was UBC and ATP5B (combined stability value = 0.056) which as with the fallopian tubes, was different to that obtained using geNorm (Table 2).

### 2.5. The Effect of Choice of Using Different Reference Genes to Normalise Data for Genes of Interest

**Figure 5:**
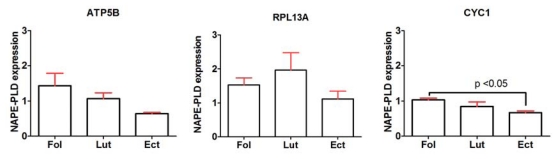

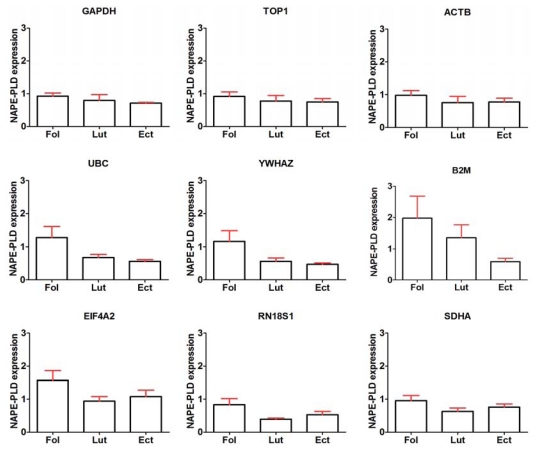
NAPE-PLD expression in human endometrium using each of the 12 candidate reference genes as internal control. Samples were from the follicular (Fol, *n* = 4), luteal (Lut, *n* = 4) phase and ectopic pregnancy (Ect, *n* = 4). Relative NAPE-PLD expression was significantly higher (*p* < 0.05) in the follicular phase than in ectopic pregnancy using CYC1 as reference gene. This statistically significant result was not demonstrated when using each of the other 11 genes as internal control though there was a tendency towards higher levels in the follicular phase than in ectopic pregnancy with most of them.

In order to test the effects of using various reference genes on the gene expression profiles, the relative expression of NAPE-PLD mRNA in the endometrium was calculated using each of the 12 reference genes as internal controls as well as using the recommended combinations of genes from both geNorm and NormFinder. The results of our analysis indicated that depending on the reference gene selected, NAPE-PLD mRNA appeared either unchanged in all three study groups (e.g., GAPDH, TOP1), attenuated in the luteal phase and ectopic pregnancy compared to the follicular phase (e.g., UBC, YWHAZ) or that expression may be increased in the luteal phase compared to the follicular phase or ectopic pregnancy (e.g., RPL13A) (Figure 5). Interestingly, there was a statistically significant increase in NAPE-PLD mRNA expression in the follicular phase compared to the ectopic pregnancy group (*p* < 0.05; one-way ANOVA) using CYC1 as the internal control. This significance was not observed when using each of the other genes as internal control (Figure 5).

### 2.6. General Discussion of Results

To the best of our knowledge, this is the first study that has evaluated the most commonly used reference genes for normalisation of RT-qPCR data in ectopic pregnancy. The absence of such a study may be due to multiple factors including the possibility that researchers may have relied on extrapolating data from similar studies in other tissues or may be selecting internal control genes without thorough validation. Many studies have shown the variation in stability of reference gene in various tissues [14,15] and we have also shown here that a reference gene which may be deemed more suitable in one tissue type may not be suitable in another tissue type even within the same cohort of patients. Justification of the choice and number of reference genes should therefore be an essential part of RT-qPCR experiments in ectopic pregnancy as recommended by the MIQE guidelines [11].

Unlike the endometrial samples, there was a wider variation in Cq values of fallopian tubes from both the ectopic pregnancy and the follicular phase compared to luteal phase, which may suggest that the local changes occurring in ectopic pregnancy may also modulate the expression of some reference genes. Arbitrary selection of reference genes is therefore not an ideal practice as the chosen reference genes may be significantly regulated in ectopic pregnancy and risk producing inaccurate data. Clearly, there was some variation in the ranking of the individual reference genes using geNorm and NormFinder. This is likely due to the different mathematical models used by both algorithms and similar findings have been reported in other studies [14,16]. Despite the differences in the ranking of the genes in fallopian tubes, the genes that were ranked in the top six by both algorithms were UBC, CYC1, GAPDH and EIF4A2 indicating that these genes are likely to be the most stable for normalisation in ectopic pregnancy studies. However, it is unlikely that a single reference gene would be an ideal internal control and the use of multiple reference genes is therefore recommended to minimise bias resulting from differential expression of one reference gene. On the contrary, a combination of genes is likely to produce more accurate results since the overall variation will be minimised. Similarly, both algorithms produced consistent results with the genes ranked among the lower half of the table *i.e.*, ACTB, SDHA, ATP5B and YWHAZ (Table 1). These genes are therefore likely to be the least suitable for use as reference genes. With regards to the endometrium, UBC and ATP5B were the only genes ranked within the top six by both algorithms suggesting that they were the most stable genes for normalisation in ectopic pregnancy studies utilising healthy non-pregnant women as control groups (Table 2). Despite the individual rankings from the endometrial samples, all the 12 genes evaluated had *M* values less than 1.5 (geNorm) indicating good stability.

In order to demonstrate the effects of using various reference genes on the gene expression profiles, the expression of NAPE-PLD mRNA in 12 endometrial samples was calculated using each of the 12 reference genes as internal control. NAPE-PLD was selected because of our group’s interest in the regulation of mammalian reproduction by the endocannabinoid system. Moreover, in a comprehensive study by Taylor *et al*, the mean immunohistochemical score for NAPE-PLD was higher in the mid proliferative compared to the mid secretory phase in both the endometrial glands and stroma [17]. The results of our analysis indicated that depending on the reference gene selected, NAPE-PLD mRNA appeared either unchanged in all 3 study groups (e.g., GAPDH, TOP1), attenuated in the luteal phase and ectopic pregnancy compared to the follicular phase (e.g., UBC, YWHAZ) or that expression may be increased in the luteal phase compared to the follicular phase or ectopic pregnancy (e.g., RPL13A) (Figure 5). Although the sample size was small, there was nonetheless a significant increase in relative NAPE-PLD mRNA expression in the follicular phase compared to ectopic pregnancy using CYC1 as the internal control. This statistically significant result was not obtained when each of the other 11 genes were used for normalisation despite a tendency towards higher levels in the follicular phase compared to the two other groups with many of the genes used (Figure 5). NAPE-PLD mRNA expression normalised to the most suitable genes as recommended by geNorm or NormFinder produced similar results with levels being higher in the follicular than in the luteal phase samples; a finding consistent with previous immunohistochemical studies (data not shown) [17]. One limitation of our study and in ectopic pregnancy research in general is the use of non-pregnant luteal phase samples as controls. However, obtaining fallopian tubes from normal pregnant women is unethical and rare in contemporary practice and coupled with the absence of a validated *ex vivo* primary human fallopian tube epithelium culture system that faithfully recapitulate *in vivo* epithelium and function, the use of these samples for ectopic pregnancy research on human subjects is likely to remain common practice. Moreover, the fact that it is in this phase of the cycle that the initial stages of embryo development, tubal transport and implantation occur make it a reasonable trade-off between what is “ideal” and what is “practical/ethical” while accepting the limitations. Despite these limitations, the results clearly demonstrate that a combination of reference genes as recommended by gene stability algorithms such as geNorm or NormFinder minimises bias that may result from variation in the reference gene itself.

## 3. Experimental Section

### 3.1. Ethics Statement

Ethical approval was obtained from the Leicestershire and Rutland local research ethics committee and all research procedures were conducted according to the principles expressed in the Declaration of Helsinki.

### 3.2. Subjects

Volunteers were recruited from the Women’s Hospital, University Hospitals of Leicester NHS Trust and were either attending as emergencies with a diagnosis of ectopic pregnancy or were non-pregnant volunteers having total abdominal hysterectomy and bilateral salpingoophorectomy (TAH & BSO) for benign gynaecological conditions such as fibroids or dysfunctional uterine bleeding. The non-pregnant were categorised into two groups based on their last menstrual period (LMP) and endometrial histology according to Noyes criteria *i.e.*, follicular phase (days 4–9) and luteal phase (days 20–25). Exclusion criteria included women with a levonorgestrel intrauterine system (Mirena^®^), on prescription or recreational drugs, or with chronic medical conditions or previous pelvic inflammatory disease (PID). Fallopian tubes and endometrial biopsies were obtained from a total of 12 volunteers [ectopic pregnancy (*n* = 4), follicular (*n* = 4) and luteal (*n* = 4)] after written and informed consent. The choice of non-pregnant women (particularly the luteal phase) as a comparison group is consistent with current practice as it is unethical and difficult to obtain fallopian tubes from women in the first trimester of pregnancy.

### 3.3. Tissue Collection

Tissue biopsies were collected immediately following TAH & BSO or following salpingectomy for ectopic pregnancy, washed (to remove blood) with sterile 1X phosphate buffered saline (PBS) and placed into sterile polypropylene tubes. Samples were immediately transported to the laboratory in liquid nitrogen and stored at −80 °C for later RNA extraction.

### 3.4. Isolation of RNA and cDNA Synthesis

Tissues obtained (50 mg) were homogenised in a mortar and total RNA extracted using Qiagen miRNeasy mini kit (50) (Qiagen, Crawley, UK) according to the manufacturer’s instructions. The RNA obtained was treated with DNAse to eliminate contaminating DNA prior to storage at −20 °C for future use. The yield and purity of isolated RNA was determined using a Nanodrop ND-1000 UV spectrophotometer (Nanodrop technologies, Wilmington, DE, USA) and samples selected for RT-qPCR had A_260/280_ absorbance ratios greater than 1.9. RNA integrity was assessed using an Agilent bioanalyser 2100 (Agilent Technologies, Wokingham, UK) and samples with RNA integrity number (RIN) of greater than seven were considered suitable for RT-qPCR.

First strand cDNA was synthesised from 1 μg of DNA free RNA using a high capacity RNA-to-cDNA reverse transcription kit (Applied biosystems, Warrington, UK) according to the manufacturer’s instructions. The final 20 μL reaction volume contained 10 μL of 2× RT buffer, 1 μL of 20× RT enzyme and 1 μg of total RNA diluted with molecular grade water to a final volume of 9 μL. A No RT reaction (RT-minus) was included to confirm the absence of contaminating DNA. The thermal conditions for reverse transcription were 37 °C for 1 h followed by 95 °C for 5 min before storage at −20 °C for future use.

### 3.5. Quantitative Real-Time PCR

Experiments were performed using the human geNorm™ reference gene selection kit containing validated lyophilised primer sets for 12 reference genes (PrimerDesign, Southampton, UK). All reactions were carried out in a final reaction volume of 20 μL containing 10 μL of 2× SYBR Green PCR Universal Master Mix (Applied Biosystems, Warrington, UK), 300 nM primer mix of reference genes or 900 nM of NAPE-PLD primers (genbank accession number NM_198990), 1 μL of cDNA and 8 μL of molecular grade water. Amplifications were performed starting with 95 °C for 10 min for enzyme activation, followed by 40 cycles of template denaturation step at 95 °C for 15 s, data collection for 60 s at 60 °C followed by extension at 72 °C. RT-minus and no template controls (NTC) containing molecular grade water instead of template mRNA were included with each run. No product was synthesised in the NTC and RT-minus confirming the absence of contaminating DNA. All reactions for the reference genes were performed in triplicate according to the manufacturer’s protocol using ABI PRISM^®^ 7000 system (Applied Biosystems, Warrington, UK). Analysis of dissociation curves for all primers showed a single peak. To calculate the PCR efficiency of primers for NAPE-PLD as the test gene of interest, standard curves were constructed which showed a linear relationship between template quantity and target gene expression. The gradient of the standard curve for the target gene was used to calculate the PCR efficiency according to the formula: Efficiency (E)(%) = (A − 1) × 100 where A = 10^[−1/slope of standard curve]^. The PCR efficiency for NAPE-PLD was 93.1% with a linear correlation coefficient (*R*^2^) of 0.972.

### 3.6. Software Determination of Reference Genes Stability

For stability comparisons of reference genes, two algorithms were used *i.e.*, geNorm version 3.5 [18] and NormFinder version 0.953 [19] according to their original publications. Data on accumulated standard deviation was obtained from the NormFinder algorithm incorporated into the GenEx software version 5.3.6.170 [20]. Details of the statistical methods used by both geNorm and NormFinder have been described elsewhere [21–23].

### 3.7. Gene Expression Stability Analysis

The mRNA expression level for each of the 12 endogenous reference genes was obtained using the mean RT-qPCR threshold cycle (Ct) value. This is defined as the number of cycles needed for the fluorescence to reach a specific threshold level of detection and is inversely correlated with the amount of template nucleic acid present in the reaction. Cq values obtained from the ABI PRISM^®^ 7000 (Applied Biosystems, Warrington, UK) output data file were converted to relative expression values using the 2^−ΔΔCq^ method [16]. Briefly,

- The highest Cq value was subtracted from all other Cq values for each gene measured. This gave a delta Cq value (ΔCq) with the highest delta Cq value being 0. All other values were less than 0.
- To each ΔCq value, the equation (2^−ΔCq^) was applied. Hence all data were expressed relative to the expression of the least expressed gene. Relative expression values were exported to geNorm and NormFinder for analysis.

### 3.8. Statistical Analysis

Except otherwise stated, data for the genes of interest were expressed as mean ± standard error of the mean (SEM) or standard deviation (SD) and p values obtained using one-way analysis of variance (ANOVA) followed by appropriate post hoc analysis using GraphPad Prism version 5.00 for Windows [24]. A *p* value of less than 0.05 was accepted as significant.

## 4. Conclusions

Altogether, our results suggest that certain genes may be more suitable as reference genes in fallopian tubes or endometrium than others. The current widespread use of RN18S1, GAPDH and β-actin in the absence of appropriate validation should be avoided. Despite the limited sample size, at the very least, it does emphasise the importance of a systematic process being needed to choose an appropriate reference gene, and that the arbitrary selection of internal controls genes may lead to misinterpretation of data.

## Figures and Tables

**Table 1:** Ranking of reference genes in fallopian tubes using the geNorm (*M* value) and NormFinder (stability value) algorithms. Lower *M* or stability value indicates more stable expression and *vice versa*. Genes highlighted in bold were those ranking consistently in the top half of the table using both algorithms.

Gene Name	Genbank Number	geNorm Ranking	*M* Value	NormFinder Ranking	Stability Value
EIF4A2	NM_001967.3	1	0.41	6	0.119
CYC1	NM_001916.3	1	0.41	4	0.109
UBC	NM_021009.5	3	0.55	1	0.098
RN18S1	NR_003286.2	4	0.59	8	0.137
B2M	NM_004048.2	5	0.65	9	0.139
GAPDH	NM_002046.3	6	0.70	5	0.115
TOP1	NM_003286.2	7	0.94	2	0.100
ATP5B	NM_001686.3	8	1.10	7	0.122
ACTB	NM_001101.3	9	1.28	12	0.154
SDHA	NM_004168.2	10	1.48	11	0.152
YWHAZ	NM_001135699.1	11	1.78	10	0.142
RPL13A	NM_012423.2	12	1.98	3	0.103
Best combination of genes		UBC, CYC1 & EIF4A2	-	UBC & TOP1	0.064

**Table 2:** Ranking of reference genes in endometrium using the geNorm (*M* value) and NormFinder (stability value) algorithms. Lower *M* or stability value indicates more stable expression and *vice versa*. Genes highlighted in bold were those ranking consistently in the top half of the table using both algorithms.

Gene Name	Genbank Number	geNorm Ranking	*M* Value	Normfinder Ranking	Stability Value
YWHAZ	NM_001135699.1	1	0.20	8	0.121
UBC	NM_021009.5	1	0.20	3	0.088
ATP5B	NM_001686.3	3	0.30	2	0.081
SDHA	NM_004168.2	4	0.39	7	0.113
RN18S1	NR_003286.2	5	0.42	10	0.144
EIF4A2	NM_001967.3	6	0.44	11	0.148
TOP1	NM_003286.2	7	0.46	5	0.106
CYC1	NM_001916.3	8	0.47	1	0.074
ACTB	NM_001101.3	9	0.48	9	0.125
GAPDH	NM_002046.3	10	0.49	6	0.106
RPL13A	NM_012423.2	11	0.52	12	0.148
B2M	NM_004048.2	12	0.56	4	0.100
Best combination of genes		UBC & YWHAZ	-	UBC & ATP5B	0.056
